# Opportunistic Rain Rate Estimation from Measurements of Satellite Downlink Attenuation: A Survey

**DOI:** 10.3390/s21175872

**Published:** 2021-08-31

**Authors:** Filippo Giannetti, Ruggero Reggiannini

**Affiliations:** Dipartimento di Ingegneria dell’Informazione, University of Pisa, 56122 Pisa, Italy; ruggero.reggiannini@unipi.it

**Keywords:** rainfall rate, rain attenuation, satellite downlink, opportunistic satellite measurements

## Abstract

Recent years have witnessed a growing interest in techniques and systems for rainfall surveillance on regional scale, with increasingly stringent requirements in terms of the following: (i) accuracy of rainfall rate measurements, (ii) adequate density of sensors over the territory, (iii) space-time continuity and completeness of data and (iv) capability to elaborate rainfall maps in near real time. The devices deployed to monitor the precipitation fields are traditionally networks of rain gauges distributed throughout the territory, along with weather radars and satellite remote sensors operating in the optical or infrared band, none of which, however, are suitable for full compliance to all of the requirements cited above. More recently, a different approach to rain rate estimation techniques has been proposed and investigated, based on the measurement of the attenuation induced by rain on signals of pre-existing radio networks either in terrestrial links, e.g., the backhaul connections in cellular networks, or in satellite-to-earth links and, among the latter, notably those between geostationary broadcast satellites and domestic subscriber terminals in the Ku and Ka bands. Knowledge of the above rain-induced attenuation permits the retrieval of the corresponding rain intensity provided that a number of meteorological and geometric parameters are known and ultimately permits estimating the rain rate locally at the receiver site. In this survey paper, we specifically focus on such a type of “opportunistic” systems for rain field monitoring, which appear very promising in view of the wide diffusion over the territory of low-cost domestic terminals for the reception of satellite signals, prospectively allowing for a considerable geographical capillarity in the distribution of sensors, at least in more densely populated areas. The purpose of the paper is to present a broad albeit synthetic overview of the numerous issues inherent in the above rain monitoring approach, along with a number of solutions and algorithms proposed in the literature in recent years, and ultimately to provide an exhaustive account of the current state of the art. Initially, the main relevant aspects of the satellite link are reviewed, including those related to satellite dynamics, frequency bands, signal formats, propagation channel and radio link geometry, all of which have a role in rainfall rate estimation algorithms. We discuss the impact of all these factors on rain estimation accuracy while also highlighting the substantial differences inherent in this approach in comparison with traditional rain monitoring techniques. We also review the basic formulas relating rain rate intensity to a variation of the received signal level or of the signal-to-noise ratio. Furthermore, we present a comprehensive literature survey of the main research issues for the aforementioned scenario and provide a brief outline of the algorithms proposed for their solution, highlighting their points of strength and weakness. The paper includes an extensive list of bibliographic references from which the material presented herein was taken.

## 1. Introduction

Real-time monitoring of atmospheric precipitations over a regional territory is an objective of primary importance for public administrators to be pursued in the context of prevention policies aimed at ensuring an adequate level of safety for people living or working in the area ([1,2,3,4,5,6], just to cite a few). In the above perspective, it is of interest to have updated rainfall maps constantly available that feature (i) good accuracy in rainfall rate (RR) measurements, (ii) space-time completeness and continuity and (iii) negligible delays in rain data provisioning so as to allow timely prediction of the rainfall distribution for any incumbent meteorological event and, when required, early adoption of adequate countermeasures to prevent or reduce the related risk.

The tools traditionally available for the estimation/prediction of rain maps belong to three categories, namely (i) a set of rain gauges distributed throughout the territory and connected to a real-time data collection network, (ii) a network of meteorological radars, (iii) deployment of weather satellites (typically geostationary, such as Meteosat satellites) for remote sensing of cloud formations and whether perturbations. However, each of these methods taken individually is not able to fully satisfy the aforementioned requirements, and their joint use for a really pervasive coverage of an entire vast region would entail formidable costs for the provisioning and deployment of (notably radar) sensors and for the infrastructure required for their integration and coordination [6,7,8].

More recently, additional “opportunistic” methods have been proposed for estimating the RR, which exploit the existence of radio links distributed throughout the area, even if installed for other purposes. Basically, these systems are of two types: (i) service or ancillary terrestrial radio links, e.g., for cellular mobile networks (specifically, backhaul links from the radio access nodes to infrastructure [9,10,11]) or those used in other fixed terrestrial networks [2,12] and (ii) radio links operating between satellites and fixed terrestrial terminals distributed throughout the territory, notably for satellite-to-home TV broadcast services provided to domestic subscribers (e.g., [1,3,4,6,13,14]).

The idea behind all these techniques is to measure the global attenuation introduced by the rain on the received signal, which is an integral function of the rainfall distribution along the radio path [15], and subsequently attempt to trace the value of RR producing this attenuation at all points of the link by means of specific inversion algorithms.

In the case of terrestrial radio links, this usually results in the use of tomographic techniques that allow estimating the rain field at a generic geographical site starting from attenuation measurements collected from different radio links that intersect one another within the vicinity of the site [16]. The resulting estimation accuracy depends on number, density and variety of directions of the available radio paths.

On the other hand, in the case of a satellite link where the radio path segment affected by the rain is very short and adjacent to the terrestrial terminal, it is often possible to achieve a relatively accurate estimate of the RR in proximity to the terrestrial receiver with a very simple inversion algorithm directly mapping the measured attenuation onto the RR estimate. These latter techniques are of particular interest since they allow carrying out as many measurements of RR over the territory as there are usable terminals. Therefore, in the presence of a large population of resident subscribers, they could prospectively offer a vast and capillary coverage of the region. For this reason, they have been the subject of considerable attention in the last couple of decades, and although initially they might have been viewed as support systems or gap fillers for preexisting rain monitoring infrastructures, later on they have been considered as a viable low-cost stand-alone measurement alternative.

With reference only to systems using satellites for RR estimation, which is the focus of this paper, a host of solutions have been proposed and analyzed that differ from each other based on the criteria used to define the numerous subsystems and parameters involved in the estimation procedure. The first to be considered here is the type of opportunistic target satellite, which obviously impacts the receiving antenna design and may require additional hardware to track the satellite position. Currently, the most mature technology in the field of opportunistic RR estimation from satellite signals relies on geostationary orbit (GEO) satellites (i.e., placed on a circular orbit laying on the equatorial plane, approximately at 35,800 km above the Earth surface). Recently, the use of Low-Earth Orbit (LEO) satellites (i.e., moving on variously-inclined circular orbits at 160–2000 km above the Earth surface, typically arranged in constellations) was proposed as well [17,18]. Among the various other aspects to be carefully considered in the RR estimator design, we mention (i) the choice of the operating frequency, impacting the sensitivity of the receiver to rain; (ii) the choice of the rain-sensitive parameter, i.e., subject to a variation in the presence of rain, to be extracted from the received waveform (either the received signal power or the signal-to-noise ratio); (iii) the selection and cost evaluation of the receiving hardware, either available commercially off-the-shelf or requiring a dedicated implementation; (iv) the development of the processing algorithm to be applied to measurements; (v) the retrieval of auxiliary information relating to the radio link geometry; (vi) the retrieval of climatic and meteorological data involved in the RR estimation algorithm; and (vii) methods to build rain maps, etc.

In this paper, we attempt to sort and classify the huge amount of material available in the literature on the above topics. We present, on the one side, a comprehensive overview of the main inherent issues to be dealt with, and we review and discuss the techniques proposed for their solution on the other side, highlighting their points of strength and weakness.

The paper is organized as follows. Initially (Section 2), the basic concepts of satellite radio communications are reviewed with particular reference to geostationary satellites, by far the most commonly used for opportunistic RR measurements, and the link budget equations are recalled for the frequency ranges used in the downlink. In Section 3, we review the various impairment factors that can reduce the received signal level in addition to the free-space loss initially (Section 3.1) in the absence of precipitations, with regard to the phenomena occurring in the propagation through the ionosphere and troposphere, and also considering the effect of station keeping maneuvers and pointing errors of the terrestrial antenna. Next (Section 3.2), we discuss some models used to predict the attenuation introduced by stratiform rain both in the melting layer and in the liquid layer, highlighting how the specific attenuation affecting the signal is related to the RR. In Section 4, we review the techniques used to measure the attenuation introduced by rain on the received signal and the elaborations needed to estimate the RR. Specifically, two widely used techniques are compared and discussed, the first based on the measurement of the satellite beacon level and the other on the evaluation of the signal-to-noise ratio of a digital payload signal. Furthermore, the analytical tools used to obtain the RR estimate from the measured attenuation are reviewed, also discussing the impact of the precipitation model and the possible non-uniformity of rain on the radio path. The substantial differences inherent in this approach in comparison with traditional rain monitoring techniques are also addressed. Subsequently, in Section 5 we provide an extensive survey of the techniques proposed in the literature for the estimation of the RR, based on measurements of attenuation of a satellite signal. The presentation is organized by sequentially addressing each of the issues inherent in this application, as pointed out above, and by briefly illustrating how the various authors deal with them and propose tailored solutions. Finally, Section 6 concludes the paper. An extensive list of bibliographic references is included from which the material presented herein was taken.

## 2. Review of Satellite Radio Links

In this paper, we consider the monitoring and utilization of the downlink signals from telecommunication satellites to fixed terrestrial receiving stations, also termed here as ground stations (GSs). The goal is the opportunistic estimation of the RR via signal attenuation measurements. Since rain attenuation significantly affects microwave satellite communication at frequencies above 10 GHz, we will focus our attention on satellite services in the Ku band and above. These bands are assigned to direct-to-home (DTH) television (TV) broadcast from GEO satellites and also to two-way broadband services, either with GEO or LEO satellites. The frequency bands allocated to these services, with reference to ITU Region 1, are illustrated in Table 1.

### 2.1. A Simple Geometrical Model of the Satellite Link through the Troposphere

**Figure 1:**
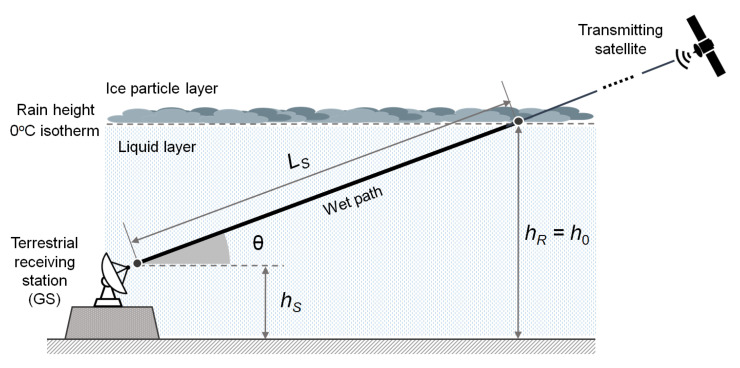
Satellite downlink geometry in the presence of stratiform rain and two-layer tropospheric model.

The downlink from a satellite to a GS in the presence of rain is shown in schematic form in Figure 1, where we assume stratiform precipitation. For now, we also assume a clear separation between two layers (tropospheric models with more layers will be addressed later). The upper layer, named “ice particles layer” (IPL), is mostly made of ice in the form of frozen dry particles, which exhibit very low scattering cross sections. This region is, therefore, associated with very small specific attenuations [23], and in the following its contribution to the attenuation of the satellite signal will be neglected. The lower layer, named “liquid layer” (LL), is instead characterized by the presence of the liquid-phase precipitation originated by ice particles that are completely melted into raindrops and introduces significant excess attenuation on the satellite signal. In such a simplified model, the boundary between the two layers, where the rain originates, has height $h_{R}$ and is assumed coincident with the height of the 0 °C isotherm, denoted as $h_{0}$.

**Figure 2:**
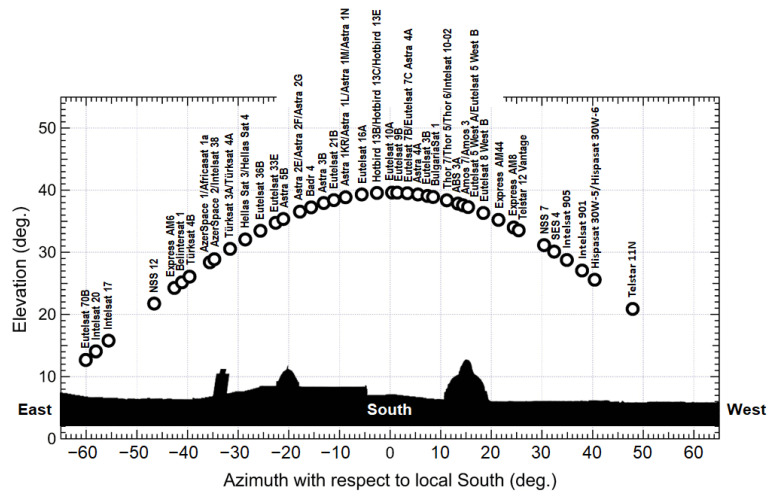
Geostationary satellite arc in the sky of Pisa, Italy (10.4147° E, 43.7117° N).

The GS is located at an altitude $h_{S}$ above mean sea level (MSL) and sees the satellite at an elevation angle θ above the horizon. The final part of the slanted electromagnetic path crosses the LL along a segment of length $L_{S}$, named the “wet segment”. For a given setting of the link parameters, the rain introduces an attenuation in an extent that depends on $L_{S}$ and on the distribution of the rain field along the wet segment. If the satellite is of the GEO type, it is observed by the terrestrial receiver under fixed azimuth/elevation (AzEl) angles, and the wet path segment also has a fixed direction with respect to a terrestrial reference. Therefore, the measurement of the rain attenuation experienced by the received signal provides an integral metric of the rain field along the wet segment. Under the assumption that the rain field is approximately constant along the wet segment (as supposed in many of the works available in the literature on the subject), the local value of the rain rate (RR) at the receiver location can be obtained from the measurement of the attenuation, regardless of the satellite position, provided that the length of the wet segments and the mapping laws between specific attenuation and RR are known (see Section 3.2 for details). If the assumption about the constant value of the rain field on the wet segment does not hold, measuring the attenuation affecting the downlink signal from a GEO satellite no longer allows achieving a fully reliable estimate for the point-scale value of RR. In this case, an alternative approach commonly pursued in the literature consists in measuring (either simultaneously or sequentially) the attenuation undergone by different signals arriving from several satellites that are in visibility relative to the geostationary arc at different AzEl angles. In this manner, the corresponding wet segments intersect the rain field along a wide variety of directions, and tomographic techniques could, thus, be proficiently applied for estimating the rain field. Table 2 enumerates all of the GEO satellites for DTH TV broadcasting in the Ku band that have at least one beam covering the city of Pisa, Italy (10.4147° E, 43.7117° N), while Figure 2 shows their positions on the orbital arc. For each satellite, Table 2 specifies the orbital longitude, along with the AzEl coordinates and the EIRP (effective isotropic radiated power) for a GS located in Pisa. This example demonstrates that many GEO satellites are potentially available for rain monitoring from a single receive site.

### 2.2. Satellite Link Budget

We now briefly review the link budget for the downlink of a GEO satellite. For the moment, we assume that the EIRP observed at the receiver site takes on the nominal value provided by the satellite operator, thus neglecting any fluctuation of the received power due to residual orbit inclination with respect to the equatorial plane, as well as to antenna pointing errors and to propagation impairments (these issues will be addressed in Section 3). Then the available power of the useful signal (also termed “carrier”) at the receiver is expressed by the following:

(1) $$C=\frac{\mathrm{EIRP}}{L_{\mathrm{FS}}L_{\mathrm{atm}}L_{\mathrm{rain}}}G_{R}$$

where $L_{\mathrm{FS}}$ is the free-space loss, $L_{\mathrm{atm}}$ is the atmospheric attenuation due to gaseous absorption (in the absence of rain), $L_{\mathrm{rain}}$ is the additional attenuation due to rain, which is the target parameter to track in order to achieve rain estimation, and $G_{R}$ is the gain of the receiving antenna of the GS. Moreover, for a digital signal with bit rate $R_{b}$, modulation alphabet size M and coding rate r (i.e, mod/cod (M, r)), the average received energy per bit and per symbol at RF is expressed by $E_{b}=C/R_{b}$ and $E_{s}=C/R_{s}$, respectively, where $R_{s}=R_{b}/\left(r\;\log_{2}M\right)$ is the symbol rate. Moreover, for both the “no rain” and “rain” conditions, the available power *N* of the additive white Gaussian noise (AWGN) at the receiver within a given bandwidth *B* (not specified here) can be expressed as follows.

(2) $$N=N_{0}B$$

where $N_{0}=k_{B}T_{\mathrm{eq}}$ is the one-sided noise power spectral density, $k_{B}$ being the Boltzmann constant and $T_{\mathrm{eq}}$ the equivalent noise temperature of the whole receiving system, given by the following.

(3) $$T_{\mathrm{eq}}=T_{A}+T_{\mathrm{RX}}$$

In (3), $T_{\mathrm{RX}}$ is the noise temperature of the receiver (assumed fixed), while $T_{A}$ is the equivalent noise temperature of the antenna given by the following:

(4) $$T_{A}=\frac{T_{c}}{L_{\mathrm{atm}}L_{\mathrm{rain}}}+T_{\mathrm{atm}}\left(1-\frac{1}{L_{\mathrm{rain}}L_{\mathrm{atm}}}\right)+T_{g}$$

where $T_{c}$ is the noise temperature of the cosmic background microwave radiation (CBMR), $T_{\mathrm{atm}}$ is the mean thermodynamic temperature of troposphere and rain (assumed to be equal) and $T_{g}$ is the elevation-dependent noise spillover temperature, accounting for the noise originated from the surrounding environment mainly from the ground and picked-up by the secondary lobes of the receiving antenna. For the sake of exemplification, Table 3 shows the values of the main downlink parameters of a DTH TV broadcast service in the Ku band and DVB-S2 format [24], with a commercial-quality receiver [8].

The quality of the received signal is expressed by the so-called signal-to-noise ratio (SNR), i.e., the ratio between the power *C* of the useful signal (see (1)) and the power of the channel noise *N* (see (2)) collected over a given bandwidth *B*, with both the powers evaluated at the output of the receiving antenna. This ratio is thus expressed as C/N and applies to any type of “carrier” signal that is either analog or digital and either modulated or unmodulated, in the latter case also termed “continuous wave” (CW). In the common case of a digitally-modulated carrier, the signal quality is more conveniently expressed by the ratio between the average energy per symbol $E_{s}$ received at RF and the noise power spectral density $N_{0}$, named energy-per-symbol-to-noise density ratio (ESNDR) and denoted as $E_{s}/N_{0}$.

## 3. Satellite Downlink Impairments

**Figure 3:**
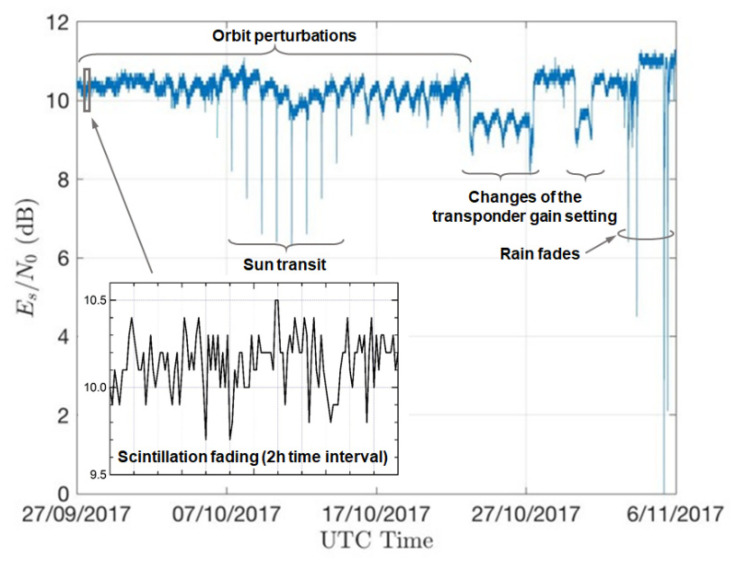
Record of received $E_{s}/N_{0}$ from the GEO satellite downlink described in Table 3 over a 6 week interval.

We hereby briefly review the main factors affecting the received signal level from a GEO satellite downlink. As examples, we refer to the measurements plotted in Figure 3, relevant to a 6 week record of the ESNDR received from the satellite link described in Table 3.

### 3.1. Attenuation Factors Affecting Signal Level without Rain (in Addition to Free Space Loss)

#### 3.1.1. Effect of Ionosphere and Troposphere

In the transit through the ionosphere and troposphere, the downlink signal is subject to scintillation fading [25], with rapid amplitude fluctuations caused by small-scale irregularities in the medium refractive index. This effect is significant for frequencies above 10 GHz and grows with frequency. In the Ku band, fluctuations are within ±0.5 dB, and the period of scintillation fades varies from 1 to 10 s. Accordingly, the spectral width of these fluctuations in the Ku band is in the order or less than 1 Hz [25]. These fluctuations are much faster than the rain events and even faster if compared to the long-term effects mentioned below (Section 3.1.2). In addition to free-space propagation loss, other attenuation factors affecting the satellite downlink are related to the presence of clouds and atmospheric absorption. The latter mainly depends on the environmental conditions (air pressure, temperature and humidity) in the vicinity of the receiving antenna, as well as on the geometry of the connection (altitude $h_{S}$ of the receiver above MSL and angle of elevation θ of the antenna) and on the operating frequency [26]. For the Ku band, it typically assumes a value of the order of a fraction of dB, with very slow variations over time, induced by the evolution of environmental conditions. Slightly larger values can be found at higher frequencies. Furthermore, the presence of clouds can affect the received signal level [27], reducing it by fractions of a dB, with slow variability over time. Finally, large-scale changes in the medium refractive index due to temperature and humidity gradients may induce downlink path bending [28]. In consequence, the received signal power may experience very slow variations. This effect, however, is significant only at very low antenna elevation angles (say around 10° or less).

#### 3.1.2. Antenna Mispointing and Orbit Perturbations

Over time, the GEO satellite is subject to many perturbing forces that render it impossible to maintain an orbit that is perfectly stable and can cause a drift both in longitude and latitude. One of the main orbit perturbations is related to the gravitational effects of the moon and the sun that cause a progression of the orbit inclination [29]. The residual orbit inclination and eccentricity cause an apparent daily movement of the satellite in elevation and longitude, as observed from the ground station, along an eight shaped path, with a 24 h period [30]. Further variations of the received signal level are caused by the slow longitudinal drift of the satellite orbital position. This is due to gravitational forces caused by earth longitudinal mass variations (for instance, satellites with longitudes from 0° to 60° East, slowly drift eastwards toward the Indian Ocean), and this sums up to the effect of orbit inclination. These perturbations are periodically counteracted by the satellite operator via orbit correction maneuvers, termed “station-keeping”: “East–West” and “North–South” station-keeping maneuvers compensate for longitudinal and latitudinal variations, respectively [31]. Thus, even if the GS antenna is correctly pointing towards the satellite nominal position, its actual gain in the link budget undergoes continuous fluctuations caused by the satellite movements, thus producing the nearly-periodic daily variations of signal strength visible in Figure 3, along with a slow decay associated to the longitudinal drift. All of the above are, however, long-term effects and their evolution is far slower than the signal fluctuations occasionally induced by a rain shower.

#### 3.1.3. Sun Blinding

Twice a year, the GS antenna pointing at a GEO satellite is occasionally “blinded” by the apparent passage of the sun behind the satellite. This phenomenon, known as “sun transit”, lasts a few minutes every day at the same time over a period of a few days. An example is shown in Figure 3, recorded around the autumn equinox in Pisa, Italy. During the sun transit, the antenna noise temperature increases abruptly and may result in severe deterioration of the SNR. However, the day/time coordinates and persistence of any sun transit can be exactly predicted, and the resulting sun-induced outage (i.e., out-of-service) events can be easily recognized and counteracted in the RR estimation procedure.

#### 3.1.4. Operator-Induced Power Variations

Occasionally, the satellite operator corrects the satellite orbital position and attitude, or changes the transponder gain setting due to customer requests or other operational needs. The above adjustment is normally carried out in a very short time, and sometimes it involves a reduction in the received power. When any such event occurs, any algorithm used to process the received data may misinterpret it as the onset of a precipitative event unless proper countermeasures are taken. A couple of such events have been observed during the measurements, as shown in Figure 3.

### 3.2. Extra Attenuation Due to Rain

**Figure 4:**
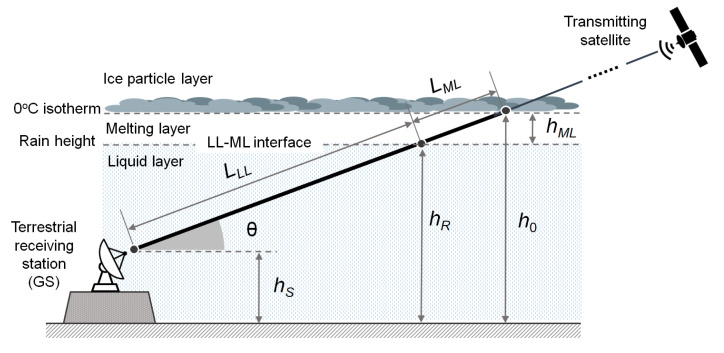
Satellite downlink geometry in the presence of stratiform rain and three-layer tropospheric model.

Figure 1 schematically shows the radio downlink between a satellite and the GS, in the presence of stratiform rain, assuming a simplified two-layer tropospheric model made of IPL and LL. As already pointed out, the rain in the LL affecting the final segment of the electromagnetic path may introduce an additional non-negligible attenuation on the received signal if the downlink frequency is close to or above 10 GHz [15] (ch. 9), while the impact of IPL is negligible. While most papers adopt the simple stratification scheme of Figure 1, some authors have investigated the more accurate three-layer tropospheric model depicted in Figure 4. Here, the lower layer containing the GS is the usual liquid layer (LL) above which there is an intermediate layer called the melting layer (ML), where the ice particles falling from the clouds gradually melt [32,33,34]. The upper edge of the ML is the freezing level (i.e., the 0 °C isotherm), while its lower edge is the level of complete liquefaction of ice, denoted as ML–LL interface in Figure 4 and located a few hundred meters below the 0 °C isotherm. The “wet segment” along the slanted path is now made of two parts: one in the LL, of length $L_{LL}$, and the other one in the ML, of length $L_{ML}$.

Taking into account the new layering model, the attenuation induced on the satellite signal by the precipitation is to be ascribed to both the liquid and melting layers, while the contribution of upper layers, above the freezing point, can be neglected. A simplifying assumption that is often formulated when dealing with stratiform rain models is that the parameters characterizing the rain field are independent of the horizontal coordinates, which implies in particular that the RR is uniform on all points of the slanted electromagnetic path through the LL. This conjecture may not always be so accurate for rain cells having size comparable or smaller than the length of the wet segment or during the initial and final phases of transit of a weather perturbation front over the receiver site.

#### 3.2.1. Specific Attenuation Model for the LL

In LL, the signal attenuation is characterized by the specific logarithmic attenuation *k* (in dB/km), which is empirically related to the RR (mm/h) by a weak exponential relationship, also termed “power-law”, such as the following [35]:

(5) $$k=aR^{b},\;\mathrm{dB}/\mathrm{km}$$

where the coefficients *a* and *b* depend mainly on carrier frequency and on other climatic, geometric and electromagnetic parameters of the connection, such as temperature, inclination of the rain with respect to the electromagnetic path and polarization. While *a* varies by orders of magnitude with the frequency in the range from 1 GHz to a few tens of GHz, *b* varies much less and does not differ very much from unity. Model (5) is assumed to be valid by all authors, even if the choice of the coefficients *a* and *b* is somewhat not uniform from author to author. Several authors (e.g., [1,6]) refer to the values of *a* and *b* recommended in [36], while others use values taken from the literature (as in [15,26]) or derived from ad hoc experiments carried out in geographical areas of specific interest [3,37,38].

#### 3.2.2. Specific Attenuation Model for the ML

With reference to a stratiform precipitation and to the model in Figure 4, the ML is defined as that layer characterized by the coexistence of solid phase (ice) and liquid phase (rain). It can be argued that the effect of the ML on signal attenuation depends both on how the liquid fraction of the precipitation varies as a function of the vertical coordinate and on the law the liquid fraction maps onto a specific attenuation value. Since there are no consolidated results/models to describe the above phenomena, various approximate approaches have been followed in the literature. One of them is to assume fictitiously that the ML is made entirely of liquid phase such as the LL so that model (5) holds for the ML as well but with a different choice for the parameters *a* and *b* [32,33,34,39]. According to this scheme, the overall attenuation is calculated as that resulting from the series of two attenuators (ML and LL) undergoing the same RR as the LL. A variant to this scheme is to retain the simplified model in Figure 1 and fictitiously modify (increase) the LL height to take into account the impact of the ML [26,36], while keeping the same values for *a* and *b* valid for the LL. Further analytical ad hoc models have been developed by other authors, e.g., in [8,40]. Additional details on these topics are given in Section 5.4.2.

## 4. Basic Methods for Estimating the Rainfall Rate from Rain Attenuation

In the following, the absence of rain, i.e., the case $L_{\mathrm{rain}}=1$ (0 dB), will be referred to as the “clear-sky” condition, and the parameters relevant to this condition will be therefore denoted by the subscript “clear”.

### 4.1. Basic Methods for Measuring Rain Attenuation

We now outline two basic schemes proposed in the literature for evaluating the attenuation incurred by the satellite signal in the downlink. The first one is based on the exploitation of the beacon signal transmitted by the satellite, while the other relies on the direct elaboration of the DVB-S/S2 signal broadcast by the satellite transponder.

#### 4.1.1. Beacon Power Measurement

**Figure 5:**
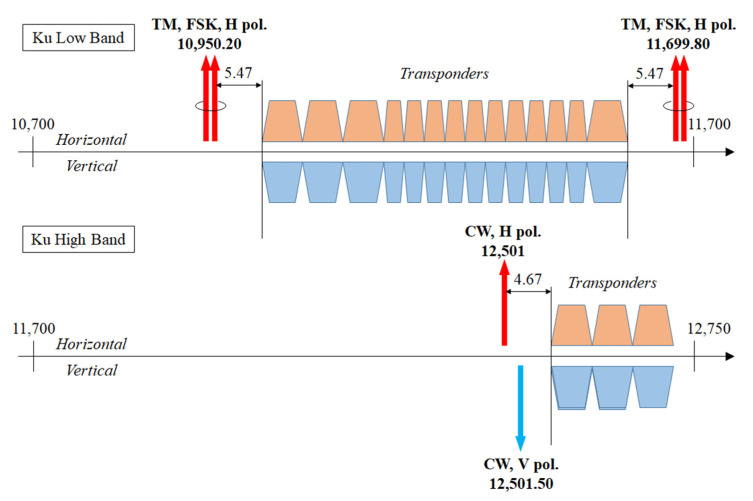
Simplified frequency plan (not to scale) of the Ku band geostationary satellite Eutelsat 10A, located at 10° East [44], showing polarization and frequency allocation of the beacons [45]. All the frequencies are in MHz. A single arrow indicates a CW beacon, while a double-arrow symbol represents a binary FSK-modulated TM beacon.

Beacons are narrow-band signals transmitted by the satellites, most often in horizontal (H) polarization, which are either unmodulated (also termed “continuous wave” or CW) and mainly intended for antenna steering and power control-purposes or digitally-modulated by FSK or PSK and carrying low-rate (i.e., a few kbit/s) telemetry (TM) data [41]. Beacons allow the operator to monitor satellite status and position, but they can also be used by customers for antenna tracking. In the case of broadcast satellites, typical values for the EIRP are 9 dBW for CW [42,43] and 4 dBW in case of TM [43]. Figure 5 (from [44]) illustrates a simplified version of the frequency plan of the Ku band geostationary satellite Eutelsat 10A located at 10° E. The arrows represent the beacons that are allocated at the sides of the frequency spectrum occupied by the transponders [45]: double-arrow symbols indicate TM beacons with binary FSK modulation, while single arrows indicate CW beacons.

Instead, the beacons radiated by communication satellites providing two-way broadband services in the Ka band to both fixed and mobile users with multiple-spot-coverage have stronger levels (typically, 30 dBW [46]). In this case, a beacon is radiated inside each beam and is used by the service customer for the initial pointing of the antenna for satellite tracking (in case of mobile reception) and for enabling uplink transmissions and power control.

**Figure 6:**
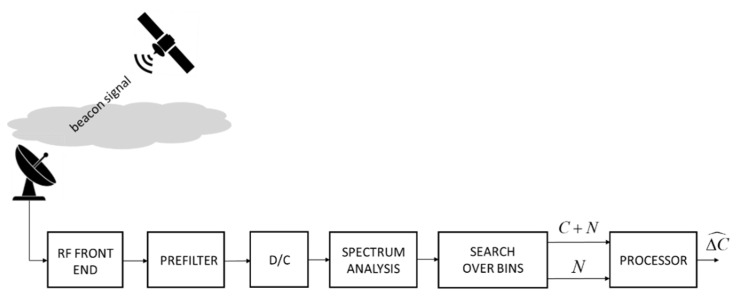
High-level block diagram of a beacon receiver for the estimate of the carrier attenuation.

Due to their constant transmit power, beacons represent an excellent reference for the measurement of the fluctuations in received signal strength caused by propagation phenomena. Figure 6 depicts the high-level conceptual scheme of the beacon-based measurement system, which was adopted by many authors [6,47,48,49,50,51].

After the RF front-end, a CW beacon is prefiltered by a narrowband band-pass filter centered around the beacon frequency. The passband is, however, wide enough (it can be in the order of magnitude of one hundred kHz) to accommodate the frequency instability of the beacon generator which is typically in the order of 1 ppm per annum and also the transmitter phase noise for which its band is only a few tens of Hz. The prefilter is followed by a down-conversion to baseband (the block “D/C” in Figure 6). Then, a vernier search in frequency of the beacon signal is carried out by means of a filter bank made, for example, with an FFT-based spectrum analyzer (the “Spectrum Analysis” block in Figure 6). The analyzer resolution is a few tens of Hz, and it is not narrower in any case than the band broadening induced by the overall phase noise of both the transmitter and the receiver. The analysis bin corresponding to the maximum level then provides a measure of the sum of the beacon power *C* and that of the noise *N* within the resolution band (i.e., the bin width), while the farthest bins (at the edges of the band) provide a measure of the noise *N* only.

From these measurements, made during both rain and clear-sky conditions, it is therefore possible to evaluate the reduction in the beacon power $\hat{\Delta C}$, representing an estimate of the rain attenuation $L_{\mathrm{rain}}$. In this respect, it is worth remarking that the presence of rain (i.e., $L_{\mathrm{rain}}>1$) has a twofold effect: on the one side, it reduces the power of the useful signal (the beacon, in this case), as in (1), while on the other side it increases the noise power, as can be inferred from (2) to (4). Therefore, it is not correct to ascribe the SNR degradation to the variation of *C* alone, although some authors seem to ignore or overlook this issue.

Recalling (1)–(4), it is possible to derive a formula for estimating the rain attenuation $L_{\mathrm{rain}}$ from measurements of the received power, both in the absence and presence of rain. The total received power within the frequency bin containing the beacon carrier (for the sake of simplicity, we assume hereafter that there is no power leakage on adjacent bins) can be expressed as follows:

(6) $$P_{\mathrm{rain}}\left(L_{\mathrm{rain}}\right)=C+N=\frac{\mathrm{EIRP}}{L_{\mathrm{FS}}L_{\mathrm{atm}}L_{\mathrm{rain}}}G_{R}+k_{B}B\left[\frac{T_{c}}{L_{\mathrm{atm}}L_{\mathrm{rain}}}+T_{\mathrm{atm}}\left(1-\frac{1}{L_{\mathrm{atm}}L_{\mathrm{rain}}}\right)+T_{g}+T_{\mathrm{RX}}\right]$$

where B denotes here the analyzer frequency resolution (i.e., the bin width); moreover, the dependence of the received power $P_{\mathrm{rain}}$ on the rain attenuation $L_{\mathrm{rain}}$ has been pointed out. Now, let us denote with $P_{\mathrm{clear}}=P_{\mathrm{rain}}(L_{\mathrm{rain}}=1)$ the received level in the absence of rain to be used as the reference for evaluating the attenuation. Then, after some manipulation, it is found that $L_{\mathrm{rain}}$ is given by the ratio between the received powers in the bin containing the beacon in the absence and in the presence of rain, respectively, where each value has to be properly corrected by the rain-independent noise power contributions.

(7) $$L_{\mathrm{rain}}=\frac{P_{\mathrm{clear}}-k_{B}B\left(T_{\mathrm{atm}}+T_{g}+T_{\mathrm{RX}}\right)}{P_{\mathrm{rain}}-k_{B}B\left(T_{\mathrm{atm}}+T_{g}+T_{\mathrm{RX}}\right)}$$

Using (7) it is possible to obtain $L_{\mathrm{rain}}$ from the measurement of $P_{\mathrm{rain}}$ and from knowledge of the reference power $P_{\mathrm{clear}}$ (the latter obtained from either link-budget analysis or previous measurements) and the temperatures $T_{\mathrm{atm}}$, $T_{g}$ and $T_{\mathrm{RX}}$ (the first being around 275 K, the latter two taken from the specifications of the receiving antenna and equipment). An alternative approach, as suggested in Figure 6, consists in jointly measuring both C+N on the bin containing the beacon power and *N* on a different bin far enough from the former containing the power peak so that only noise can be safely measured there. The difference between the powers in the bin containing the peak and in the noise-only bin yields the value of *C*. This procedure is applied twice, i.e., for both the clear-sky and the rain condition, and the ratio between the values of *C* obtained in the two conditions will again yield $L_{\mathrm{rain}}$.

For the sake of clarity and in order to demonstrate the potentials of the beacon-based method, let us consider the scenario described in Table 4a where a high-power beacon signal from a Ka-band broadband service satellite is exploited, and the parameter values are based on those used in [47]. The resulting performance, in both clear-sky and rain conditions (with $L_{\mathrm{rain}}$ = 44 dB, corresponding to a rain rate in excess of 100 mm/h over wet paths of 3–4 km at 20 GHz (see e.g., Figure 9.7 in Ref. [15]), is presented in Table 4b. In particular, it is shown that even in this case of torrential rain, the beacon power still exceeds by around 5.4 dB the background Gaussian noise power measured within the frequency bin, thus allowing reliable detection and estimation of the signal peak level.

As pointed out earlier, these kinds of devices are mainly proposed for systems operating in the Ka band or higher, where more powerful beacons are available [6], and seldom for the Ku band. Since COTS (i.e., commercially available off-the-shelf) devices are difficult (if not impossible at all) to find for beacon reception, practical implementations must substantially rely on ad hoc self-made receivers. However, this additional burden is likely to pay off since, thanks to the frequency stability of beacon generators and to the consequent high resolution of the spectral analysis, these devices allow, as shown above, measuring variations of *C* over dynamic ranges spanning several tens of dB [47]. Therefore, they allow estimating very high values of RR (up to more than 100 mm/h).

#### 4.1.2. SNR or ESNDR Measurement on a Wideband Signal

**Figure 7:**
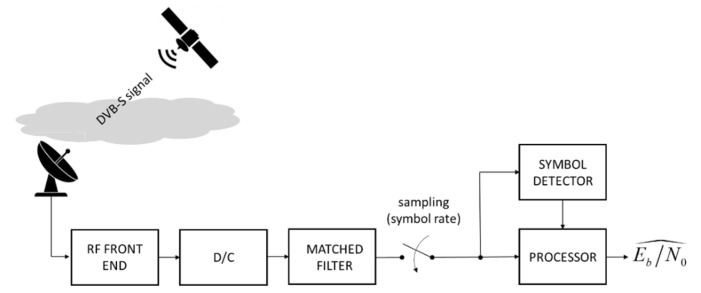
High-level block diagram of a broadband signal receiver (e.g., DVB-S) for the estimate of the ESNDR.

In many other papers, the estimate of the RR is based on measurements made by the receiver directly on the payload signal. This is the typical case of commercial-grade receivers for the reception of TV signals in DVB-S/S2 format broadcast in the Ku band [8] or for the download streams of internet access systems operating in the Ka band or higher [5]. In these cases, the conceptual scheme of the measurement system is that of Figure 7 in which the payload signal is processed using a classic receiver chain (i.e., front end, downconversion, matched filter and sampler). The noise-corrupted samples at the output of the matched filter are sent to an algorithm that compares them with the noise-free symbols at the output of the symbol detector and provides an estimate of ESNDR ($E_{s}/N_{0}$) under the assumption that the decisions on symbols are error-free. The system analyzed in [8] features an innovative device for domestic reception of satellite TV broadcasts in the Ku-band, which is capable of continuously generate estimated values of $E_{s}/N_{0}$ and possibly retransmit them back on a return satellite channel either in the Ku or in the Ka band (more details are provided in Section 5.2.2). Another instance is in [5], discussing a specific implementation of the terrestrial terminal equipped with an estimator of the SNR. Although details of the latter are missing, it can be argued that its operation is similar to the one in [8]. In both the above papers, the proposed systems use a return channel that allows concentrating the SNR data relevant to the received signals from the various GSs to a single centralized control station. In [8], it is shown that, for a digital transmission with symbol rate $R_{s}$, the ESNDR can be written as a function of the rain attenuation as follows.

(8) $${\left(\frac{E_{s}}{N_{0}}\right)}_{\mathrm{rain}}(L_{\mathrm{rain}})=\frac{\mathrm{EIRP}}{k_{B}R_{s}L_{\mathrm{atm}}L_{\mathrm{rain}}\left[\frac{T_{c}}{L_{\mathrm{atm}}L_{\mathrm{rain}}}+T_{\mathrm{atm}}\left(1-\frac{1}{L_{\mathrm{atm}}L_{\mathrm{rain}}}\right)+T_{g}+T_{\mathrm{RX}}\right]}$$

Denoting the ESNDR in clear-sky conditions as ${\left(E_{s}/N_{0}\right)}_{\mathrm{clear}}={\left(E_{s}/N_{0}\right)}_{\mathrm{rain}}(L_{\mathrm{rain}}=1)$, we obtain the following expression for the rain attenuation:

(9) $$L_{\mathrm{rain}}=\frac{{\left(E_{s}/N_{0}\right)}_{\mathrm{clear}}}{{\left(E_{s}/N_{0}\right)}_{\mathrm{rain}}}\left(1-\xi \right)+\xi$$

where we defined the following.

(10) $$\xi =\frac{T_{\mathrm{atm}}-T_{c}}{L_{\mathrm{atm}}\left(T_{\mathrm{atm}}+T_{g}+T_{\mathrm{RX}}\right)}$$

This type of approach can be easily pursued since the market offers low-cost COTS receivers capable of performing SNR or ESNDR estimates, and some of them are also equipped with a return channel, as noted earlier [5,8].

### 4.2. Mapping of Rain Attenuation onto Rainfall Rate and Related Issues

#### 4.2.1. General Procedure for Mapping

As previously pointed out, the attenuation $L_{\mathrm{rain}}$ introduced by the rain on the received signal is an integral value depending on the particular distribution of the rain field along the “wet segment” of the satellite–GS radio path (see Figure 1). This implies that, in general, the knowledge of $L_{\mathrm{rain}}$ alone is not enough for the retrieval of the RR in the proximity of the receiver. However, if we add the assumption that the RR is approximately constant on the aforementioned wet segment, the problem of estimating RR at the receiver can be easily solved provided that the length of the wet segment is known. Actually, by dividing $L_{\mathrm{rain}}$ (expressed in dB) by the length $L_{s}$ of the wet path (in km), we find the specific attenuation *k* (in dB/km):

(11) $$k=\frac{L_{\mathrm{rain}}}{L_{s}}\;(\mathrm{dB}/\mathrm{km})$$

which is expressed by (5) as a function of the RR. In the simple precipitation model of Figure 1, the length of the wet path can be obtained from the elevation angle θ of the GS antenna and the rain height with respect to the level of the GS, $h_{R}-h_{S}$, is as follows.

(12) $$L_{s}=\frac{h_{R}-h_{S}}{\sin\theta }$$

Moreover, assuming that the coefficients *a* and *b* are known, the inversion of (5) eventually yields an estimate of the RR.

#### 4.2.2. Melting Layer Effects

A more accurate RR retrieval procedure can be implemented by specifically considering the impact of the ML. Recalling the discussion in Section 3.2, a possible approach is to adopt the precipitation model illustrated in Figure 4 and to ascribe to the LL and to the ML separate contributions to the overall attenuation $L_{\mathrm{rain}}$, both based on power-laws as in (5) when expressed in logarithmic form, but with coefficients specifically calibrated for each layer. The global (logarithmic) attenuation is the sum of these two contributions. When following this procedure, the two cited contributions summing up to $L_{\mathrm{rain}}$ must be expressed as a function of the RR present in the LL, and subsequently the relationship must be inverted in order to provide the RR corresponding to a global measured logarithmic attenuation (see [8] (Section V) for an example). Alternatively, we could retain the simple single-layer model in Figure 1, where the height of the rain is fictitiously modified by purposely adding a corrective term to the 0 °C isotherm height as recommended in [52]. In this manner, we are reverted to a single-layer model, but with an extended wet segment, which produces an additional attenuation equivalent to that of the ML. In this case, the inversion procedure is facilitated as we have to deal with a single power-law term. Some further details on these issues are discussed in Section 5.4.1 and Section 5.4.2.

#### 4.2.3. Non-Uniformity of the Rain and Geometrical Issues

**Figure 8:**
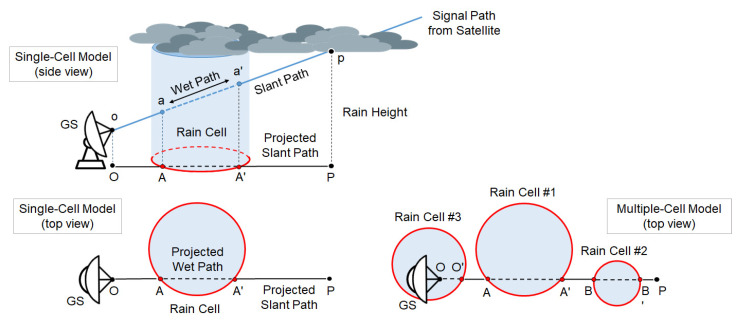
Precipitation modeled as one or more cylindrically-shaped rain cell(s) with uniform RR inside.

The assumption of uniform rain distribution along the whole length of the wet segment may actually prove unrealistic in many cases. Even if we model the precipitation field structure as one or more spatially-separated, cylindrically-shaped rain cell(s) with uniform RR inside (as shown in Figure 8), the intercept of the cell(s) and the slant path $\bar{op}$ will typically affect only a part of the path itself. The problem of a possible non-uniformity of the rain field along the wet segment is tackled in [53], where the accuracy of the RR estimate at the receiver, obtained from a single-layer model, is improved by applying a corrective coefficient to the value of $L_{\mathrm{rain}}$. In addition to the usual geometrical parameters of the link (i.e., length of the wet segment and antenna elevation angle), this coefficient also takes into account the following features:

- The diameter of a single (cylindrically-shaped) rain cell;
- The average length of the intercept of the projected slant path with the randomly positioned circles representing the ground projection of multiple rain cells;
- A calibration coefficient which takes into account the non-uniform distribution of rain inside the cell.

## 5. Survey of Rainfall Rate Estimation Techniques

Many opportunistic techniques for RR estimation by means of satellite receivers are available in the literature. As already mentioned, the various techniques are characterized by a variety of solutions and features that will be addressed hereafter in greater detail, namely with respect to the following:

(i) Satellite orbit and downlink frequency band (Section 5.1);
(ii) Physical quantity to measure, generically termed as the “strength” of the received signal, and to be processed for estimating the RR and the type of receiver (Section 5.2);
(iii) Type of processing (Section 5.3) for the following:
  - The detection of a rain event, together with the relevant start and end epochs;
  - Identifying the “baseline” signal strength in the absence of rain (“clear-sky”) to be used for evaluating the signal strength reduction during rain events;
  - Obtaining the estimate of RR from the measurement of signal strength reduction;
(i) Ancillary information (geometric, climatic and meteorological) required by the RR estimation algorithm and how to collect it (Section 5.4);
(ii) Techniques for recognizing and tackling sudden strength variations of the signal strength, not due to rain (Section 5.5).

### 5.1. Type of Satellites

#### 5.1.1. Satellite Orbits and Frequency Bands

The vast majority of the systems cited in the bibliography refers to the use of one or more GEO satellites, but some solutions have also been proposed, especially in recent times, for the case of LEO satellite constellations belonging to global mobile communication networks [17,18]. With reference to Table 2, the downlink frequencies associated with the GEO broadcasting satellites are mostly in Ku band, plus a minor slot in the Ka band, while broadband communication satellites, either for fixed (GEO) and mobile (LEO) services, operate exclusively in the Ka band. In the recent years, the growing demand for higher throughput stimulated research and experimentation in the spectrum above 30 GHz (the Q/V band) [54,55]. From the perspective of the opportunistic approach relative to rain measurement, this migration from Ku to Ka and then to Q/V is a really appealing opportunity because the higher the frequency, the greater the effect of rain attenuation and the more easily it can be measured.

#### 5.1.2. Tomographic Approaches and Other Techniques for the Reconstruction of the Precipitation Fields

A GEO satellite is observed from terrestrial terminals under a constant azimuth angle, which depends on the (fixed) longitude of the satellite. Consequently, in order to probe the rain field along other directions, it is necessary to resort to the joint reception of several GEO satellites positioned over a sufficiently wide arc of longitudes [50,54,55,56]. Tomographic techniques have been, therefore, proposed for estimating the rain fields on a territory covered by several geographically distributed receivers and/or by using multi-antenna GSs capable of simultaneously receiving at the same site several satellites visible on different azimuth and elevation angles. This type of techniques can, in principle, provide the most accurate estimation of non-uniform rain fields at ground level. In case of LEO constellations, the mobility of the satellites across the sky instead allows probing the volume affected by the rain in several directions; therefore, it lends itself to the use of tomographic techniques much more easily than for GEO [17,54,55]. In fact, a terrestrial terminal observes each single LEO satellite for scanning a wide range of azimuth angles as the satellite moves (except in the particular case in which the satellite ground track passes exactly on the point where the terminal is located; in this case, the terrestrial terminal observes the satellites under a constant azimuth and with an elevation from 0° up to 90° and then down again to 0°).

It is worth remarking that all of the currently available prototypes operate with GEO satellites only, while the utilization of LEO signals remains thus far at the level of feasibility studies; therefore, they will not be further pursued in the following.

### 5.2. Measurement of Signal Level at the Receiver and Related Hardware Issues

All the proposed RR estimation algorithms are based on the monitoring of a performance measure of the received signal, such as the signal power level or the SNR or the ESNDR, and on the evaluation of its reduction caused by rain compared to clear-sky conditions. The knowledge of this reduction in the performance measure allows evaluating the attenuation introduced by the rain on the radio link and eventually estimating the corresponding RR.

#### 5.2.1. Beacon-Based Approach

One of the most used techniques consists of measuring the level of the unmodulated beacon signal transmitted by the satellite and evaluating the attenuation due to rain by means of the procedure outlined in Section 4.1.1, where Figure 6 details the required signal processing. As discussed in Section 4.1.1, this technique allows obtaining a good measurement dynamic range thanks to the possibility of using a very narrow band filter for the detection of the beacon signal. In this manner, in clear-sky conditions, it is possible to obtain very high SNRs (tens of dB) which enable a wide dynamic range in the measurement of the rain attenuation and in the estimates of RR (many tens mm/h or more). The use of higher frequency bands, such as the Ka or the Q band, can further improve the sensitivity of the algorithm to rain as demonstrated in [50,54,55].

Some of the solutions available in the literature produce estimates of the RR directly from the measurement of the variation of the overall received power, apparently ignoring the effect of the rain noise (for example [50,54], they seem to adhere to this simplified approach).

Most works instead illustrate, in detail, the whole signal processing chain, starting from the measurement of the total power at the receiver prefilter output and producing the estimate of the beacon power after removing the effect of noise (e.g., see the discussion in Section 4.1.1). The latter approach is expected to yield better accuracy at low SNR values, especially when the attenuation due to rain (and therefore the impact of the relevant noise) is severe.

A common feature of beacon-based RR estimators is the need of a custom hardware realization of the beacon receiver/meter device, which can render this approach quite onerous especially if compared with COTS-based solutions (see hereafter). This raises serious doubts about the feasibility of a wide network of sensors of this type, with a reasonable effort.

#### 5.2.2. COTS-Receiver-Based Approach—Ku Band

A promising and cheaper alternative relies on low-cost COTS terminals, commonly known as set-top boxes (STBs), which are already installed in millions of domestic premises worldwide for the reception of DTH TV broadcasts from GEO satellites, mainly in the Ku band. STBs are typically receive-only devices, and the return channel, when available, reaches the broadcaster only via the internet network. Any STB has some signal strength meter functionality for system set-up, parabola aiming and diagnostic purposes which could, in principle, provide useful material for RR estimation. Unfortunately, accessing these data may reveal a harsh (if not impossible at all) task for most of the COTS terminals. The situation is much easier in case of “open” receivers, i.e., based on the open-source Linux operating system where a skilled user, via ethernet interfacing, can upgrade the firmware and read the receiver internal status, including the received signal strength. Furthermore, a next-generation of interactive satellite terminals has been recently launched in the market and could gradually replace the equipment currently employed for satellite TV reception. This novel device, commercially branded by Eutelsat as “SmartLNB” [8], has the appearance of a common LNB (Low-Noise Block, i.e., the outdoor unit mounted in the focus of the parabolic reflector), but it exhibits some remarkable innovative features, namely the following: (a) It is only slightly larger than a conventional LNB, but it integrates within its case all functions of both the LNB and the decoder (i.e., the indoor unit which is placed next to the TV set or directly integrated in it), thus representing a complete and compact DTH satellite receive system; (b) it performs an accurate real-time monitoring of the received signal quality by producing frequent estimates of the ESNDR (typically, one estimate per minute), as shown in Figure 7; (c) it is a two-way device that includes also a low-power transmitter enabling a low-data-rate packed-based return channel, i.e., an RF link from the user premises (in the Ku or Ka band), via the same GEO satellite to a Service Center (SC) in charge of collecting and processing user data [57] for pay-per-view, social networking, live interaction, subscription management, audience meter and IoT (Internet of Things) services.

The features in (b) and (c) make the SmartLNB a very appealing satellite receiving equipment for opportunistic RR measurements. Actually, the ESNDR readings are encapsulated, together with other information about the device status, into the return channel data stream and, without the need for any terrestrial communication infrastructure, are sent via satellite to the SC where they can potentially feed the RR estimation algorithm. Thus, thanks to both its embedded ESNDR estimator and built-in transmitter, any SmartLNB for domestic satellite reception can also be observed as a signal strength meter equipped with a modem for wireless data collection, and this works from any location within the area covered by the GEO satellite. Furthermore, a single GEO satellite for TV broadcasting typically serves many countries or even a whole continent; for instance, Eutelsat’s Ku band satellites located at 10°, 13° and 16° East cover all the European continent (plus surrounding areas such as North Africa, the Mediterranean basin, the Anatolian Peninsula and inner Russia). This means that any GEO satellite has a potential audience of several millions domestic DTH receivers that are, prospectively, also all measurement sites. The possible collisions among the huge number of packets arriving at the SC via the return link are effectively tackled and solved thanks to a high performance Random Access protocol dubbed Enhanced Spread Spectrum Aloha (E-SSA), especially conceived for packet-based return link in satellite applications and featuring Successive Interference Cancellation (SIC) techniques. It is shown that, due to jointly a robust rate in the turbo encoder and to the iterative cancellations, the packet collisions do not affect the system performance [58].

This not enables the construction of rain maps, reasonably on regional, but also on an even broader scale where each terminal corresponds to a point on the map and for which the spatial resolution is related to the density of terminals installed on the territory.

An approach based on Eutelsat’s SmartLNB receivers was studied in the framework of the Nefocast project (funded by regional administration of Tuscany, Italy), as discussed in [3,8].

Once the ESNDR readings are somehow made available for processing, either using properly-interfaced open STBs or SmartLNBs, the RR estimation algorithm operates in the same manner. That is, the ESNDR is continuously tracked and compared with a clear-sky reference level, which can be prestored at the initial setup or adaptively upgraded from previous days measurements. When a rain fade is detected, the corresponding attenuation is analytically derived from (9). Then, the specific rain attenuation *k* (in dB/km) in the LL can be obtained by resorting to the tropospheric model in Figure 1, and the instantaneous RR value is eventually achieved by inverting the power-law in (5). Alternatively, the effect of the ML can be taken into account, by means of improved precipitation inversion models, such as that in [8], which is based on the three-layer model in Figure 4 or that in [40].

**Figure 9:**
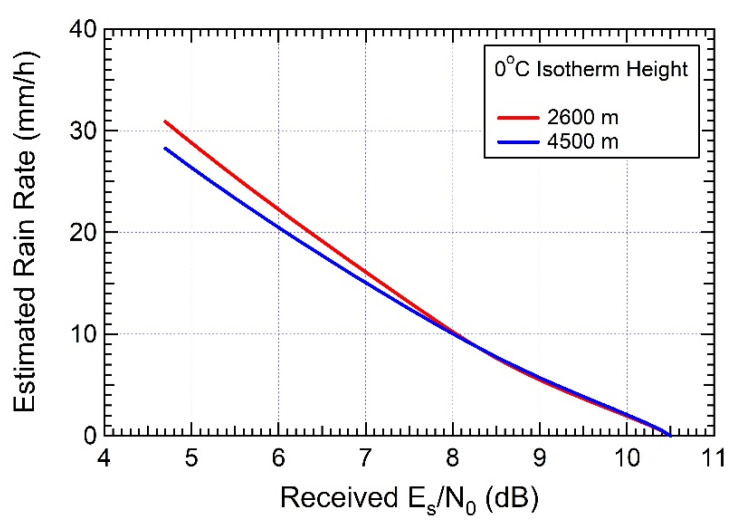
Dynamic range of the RR estimation technique in [8], with 10.5 dB of nominal clear-sky ESNDR and 4.68 dB of minimum required ESNDR (see Table 3).

A possible point of weakness for a system of this type is related to the dynamic range it offers for the measurement of the ESNDR. Actually, the rain margin of these satellite downlinks is tailored for the peculiar service they are intended towards (i.e., TV broadcast or broadband internet) such that that intense precipitations may cause service outage with a given non-negligible probability set by design specifications. Accordingly, in a practical implementation of the system, the nominal value of ESNDR at the receiver with standard reception equipment and in clear-sky conditions is typically chosen with a few (say 5–6) dB of margin above the minimum required threshold ESNDR (set by the selected signal mod/cod, see Section 2.2), below which the receiver incurs outage. For instance, the experimental setup described in [8] uses the downlink of the EUTELSAT 10A satellite with a link margin of around 5.8 dB (see the parameters in Table 3). Most of the receiver ESNDR estimators rely on carrier and clock synchronization; therefore, during the outage caused by heavy rain, they are unable to provide any useful estimate (however, there are also some receivers that are capable of estimating values of ESNDR below the mod/cod threshold). Although tolerable for the intended broadcast or broadband services, link breakdowns inhibit the detection and measurement of the most intense precipitations, which are instead the most interesting ones from the perspective of a rain monitoring system. The resulting dynamic ranges offered by COTS devices for estimating rain attenuation turn out to be limited to a few tens of mm/h, as confirmed by Figure 9 which refers to the algorithm described in [8] and presents the estimates of the RR vs. the ESNDR within the receiver operating range, from 4.68 dB (minimum required value for the selected mod/cod) to 10.5 dB (nominal received level in clear-sky conditions). As is apparent, even with some sensitivity to the value of the 0 °C isotherm height, the maximum RR that can be measured is around 30 mm/h. In order to provide an idea of what this value represents in terms of probability, let us consider the table of rainfall intensity for the world rain climatic zones in ITU-R recommendation 837-1 [59] (we refer to this superseded recommendation for the sake of exemplification only). In region “L”, which includes Western Tuscany (Italy) where the experiments of [8] have been carried out, the RR is shown to exceed 33 mm/h with a probability of 0.03%. This means that, during one year, the received ESNDR falls below the threshold 4.68 dB for approximately 160 min, i.e., 2 h 20′, overall, and this is also the total duration of outages which could inhibit RR measurements.

#### 5.2.3. COTS-Receiver-Based Approach—Ka Band

A system similar to the previous one and operating in the Ka band is proposed in [5], where a method is discussed allowing the estimation of RR from measurements of the received SNR taken at constant rate (one every 5′) on a broadband payload signal transmitted by an existing broadcast satellite. The paper does not provide details on how the SNR observations are mapped onto values of rain attenuation undergone by the satellite signal, nor does it describe the relationship existing between the SNR and the RR. In particular, it provides no hints on how the noise power is related to rain intensity. In any case, [5] confirms that the SNR margin available for rain estimation is very narrow, since the baseline SNR is designed so as to guarantee a satisfactory quality of the received signal in the nominal dry operating conditions, with an edge of only a few dB over the outage limit. Consequently, the range of measurable RRs is very small and hardly exceeds 10 mm/h. As discussed in Section 4.1, a viable and reliable route for achieving a wider dynamic range for signal attenuation is to resort to systems measuring the level of calibrated narrowband beacon sources.

### 5.3. Processing Issues

The performance of a RR estimation algorithm based on measurements of downlink SNR or ESNDR is strictly related to the specific criteria utilized to implement the numerous functions and processing steps involved in the algorithm. We hereby provide a short overview of the relevant issues.

#### 5.3.1. Identification of the Rainy Periods

A particularly crucial issue is the identification of the starting and ending instants of the time intervals within which the received signal incurs a level reduction actually due to the presence of rain on the satellite–GS radio path. The problem cannot be solved in a trivial manner in view of the large number of possible rain-independent factors that can cause a drop in the signal level, as discussed in Section 3.1, and a good algorithm should be able to distinguish the effect of rain from those imputable to other causes.

In general, all algorithms proposed in the literature are designed to be insensitive to fast fluctuations due to ionospheric scintillation, as the raw SNR or ESNDR measurements are usually smoothed out at some early processing stage by a low-pass filter calibrated on the time variability properties of the rain attenuation process, which is a far slower phenomenon than scintillation. Moreover, in order to reliably recognize the rain-induced signal level variations, it is necessary to identify and compensate for the slow signal fluctuations deriving from oscillations and drifts of the satellite position or from power/attitude corrections (Section 3.1.2), which can be misinterpreted as rain effects. We return on these issues further below.

The techniques devised to recognize the start and stop instants of a rain event are numerous and characterized by different levels of complexity. A straightforward albeit rather simplistic approach involves the preventive estimation of the dry baseline level by averaging measurements of SNR or ESNDR over very long time intervals (order of days), followed by the choice of a rain detection threshold, adequately lower than the above dry baseline to reduce the risk of false detections (normally at least 1 dB down). The presence of rain is declared when the sequence of measurements drops below this threshold, and conversely the dry condition is assumed when it is above. The baseline level can be continuously updated offline during dry periods. In order to limit the incidence of false rain detections, the distance between the baseline and the threshold must not be affected by the slow SNR/ESNDR fluctuations mentioned above, which can significantly reduce the sensitivity of the algorithm, making it unable to detect small intensity rains. Furthermore, depending on how the low-pass filter for smoothing out fast fluctuations is designed, a delay in rain detection may be introduced where it is not always compatible with the real-time response requirement of certain applications. For instance, in [1,6,49], sliding window filters are used with response latency in the order of tens of minutes.

In [3,8], the dry baseline against which to compare the series of SNR or ESNDR values is updated in real time through the use of a “slow” Kalman tracker, featuring a very long time constant in comparison with the typical length of fluctuations induced by rain events (and even more so with respect to scintillation). Its output during dry periods faithfully reproduces the trend of very slow fluctuations such as those due to satellite oscillations and station keeping maneuvers. In parallel to this device, a second Kalman tracker operates with a faster response which, while removing the fluctuations due to scintillation, is able to track with negligible delay the variations of SNR/ESNDR at the occurrence of a rain event. Therefore, while in the dry state the two trackers have almost coincident outputs, a rain onset event produces a sudden deviation (in reduction) of the fast tracker output compared to the slow one which, due to its inertia, remains insensitive to rain for a few minutes. Conversely, the end of rain is identified by the fast tracker returning close to the baseline level, the latter determined during the wet periods as discussed in the next section (Section 5.3.2). Due to this “adaptive” nature of the algorithm, the distance of the rain detection threshold from the baseline can be reduced down to a small fraction of a dB (e.g., 0.3 dB); therefore, the algorithm allows a sensitivity gain compared to the systems described earlier.

A different and more elaborate approach [5] consists of modeling the slow daily fluctuations affecting the SNR/ESNDR in the dry state as sinusoidal (see the example in Figure 3), followed by estimating the parameters of this sinusoid and eventually proceeding to its preventive cancellation from the sequence of observations. The purpose of this procedure is to obtain, after the above cancellation, a substantially constant dry baseline against which to measure the deviations of the current SNR/ESNDR during rainy periods. The latter are recognized in [5] by means of a neural network operating on the basis of the different statistical behavior (notably the more pronounced fluctuations) exhibited by the SNR/ESNDR time series in rainy periods compared to dry conditions. The cited paper does not discuss how the RR estimates are impacted whenever the aforementioned sinusoidal model fails, for example, during station keeping adjustments. Furthermore, the need to previously estimate and remove the periodic fluctuation from the input data seems hardly compatible with the algorithm capability to operate in real time.

In addition [5], other authors [1,40] have investigated the use of an appropriately trained neural network to identify rainy periods, geared to analyze, recognize and classify the statistical behavior of the received signal. This category of techniques appears promising even though, at the moment, it does not seem to clearly demonstrate that it is definitely more advantageous than the other more traditional methods, particularly with regard to the accuracy achievable in identifying the instants of start and stop of a rain event. Furthermore, [8] proposes a technique having the same purpose as the neural network in [5], although conceptually simpler. In fact, in [8], immediately after the detection of rain by means of the double slow/fast Kalman tracker mentioned earlier, a confirmation of this rain event is sought by estimating the dispersion of the current SNR/ESNDR fluctuations around their average and then, on the basis of these statistics, making a decision about the actual presence of rain.

Finally, we mention the approach adopted in [4,7] to recognize rainy periods based on a metric related to the receiver bit error rate (BER), a parameter provided by most domestic satellite receivers. Here, the underlying idea is that since the receiver BER is univoquely related to the current SNR/ESNDR, it is possible to infer the presence of rain by monitoring the BER. However, this technique leaves some doubts about the sensitivity in detecting small RRs and also in the ability to counteract the slow variations of signal level discussed in Section 3.1.2.

#### 5.3.2. Identification of the Dry Baseline during Dry and Rainy Periods

The problem of identifying the dry baseline to be used as reference level against which to measure variations of SNR or ESNDR must be addressed differently depending on whether we assume carrying out the above identification in the absence or presence of rain. In the former case, it is sufficient to accurately track the SNR or ESNDR (and its possible evolutions, as discussed Section 3.1), waiting for a rain event to occur. This task appears fairly straightforward since, in the absence of rain, the desired baseline value is “embedded” in the received signal as actual SNR or ESNDR, and it is sufficient to perform a conventional estimation of this parameter minimizing the effect of noise and of the other occasional impairments mentioned in Section 2.1.

Conversely, during a precipitation event, the dry baseline SNR or ESNDR is no longer embedded in the received signal, making it necessary to figure out the hypothetical value that would be taken on by this parameter if it did not rain. This parameter is crucial for the algorithm operation since it serves as reference level against which to assess the attenuation induced by rain. Obviously, the latter task is more difficult than the former, and its solution necessarily passes through a sort of “guess” of the dry baseline, as this parameter is not observable during rain.

Furthermore, while this guess is certainly accurate at the very start of the rain event when the dry baseline cannot deviate much from the value assumed immediately before rain inception, conversely over a long period of uninterrupted rain (say in the order of tens of minutes and more) the actual current (unknown) dry baseline may depart significantly from its initial value; therefore, it becomes appropriate to try to figure out what its actual evolution would be by using all the available a priori information about its behavior.

As for the determination of the dry baseline in the dry periods, in the previous section we have already provided a review of the main techniques proposed in the literature to carry out this task [1,5,8,49]. They differ from one another mainly for the various approaches devised to counteract the slow fluctuations affecting the measurements of the performance parameter under the dry state assumption.

Turning to the determination of the dry baseline in rainy periods, there is a variety of approaches proposed in the literature. As already mentioned, some authors [1] use a fixed or very slowly varying baseline during both dry and rainy periods, obtained from a long-term average of dry-state measurements. This type of approach is conceptually simple but not very accurate since in order to avoid false rain detections, the chosen reference level has to cut out the daily fluctuations due to the satellite motion (Section 3.1); therefore, it is hardly sensitive to small RRs. Furthermore, in [5] where, as already obesrved, the dry baseline is obtained by canceling the periodic daily component from the series of SNR measurements taken in the dry periods, at rain inception this baseline is “frozen” and used as a constant reference for the entire rain event, regardless of its duration and without taking into account any fluctuations in the dry reference that may occur during the rain. This approach may in fact result in growing errors as the rain event goes on.

In other cases [5,54], the dry baseline during rainy periods is obtained by linearly interpolating between the two measured dry levels immediately preceding and following the rain event. It is observed that this type of offline approach is not compatible with real-time operation of the RR estimator since the aforementioned interpolation of the baseline requires that the end of rain is waited for.

A further variant is in [3,8], where at the onset of a rainy period the last dry baseline provided by the aforementioned slow Kalman tracker (Section 5.3.1) is used as the initial reference, thence the slow Kalman tracker is stopped and the RR estimation algorithm proceeds by using as reference the dry baseline values utilized the previous day at the same time that have been stored in memory. The data drawn from the previous day are corrected by adding a fixed shift (accounting for a possible long-term drift of the baseline) so as to fit them to the dry-state measurements available up to the rain event, avoiding any discontinuities. In doing so, the most likely inherent daily fluctuations of the baseline are taken into account even while it rains, improving the real-time operation of the algorithm.

#### 5.3.3. Rainfall Rate Estimation and Comparison with Rain Gauges

All algorithms proposed for RR estimation pass through the determination of the specific attenuation *k* (dB/km) characterizing the wet segment of the satellite-GS path and related to RR by (5), where the values of the coefficients *a* and *b* are determined as discussed in Section 3.2.1. Several authors [3,5] adhere to the recommendations [36] which provide typical values for *a* and *b*, while others employ values deriving from dedicated experimental campaigns carried out in specific areas of interest, possibly with the use of disdrometers [37,60] and are, therefore, to be considered more accurate for the above geographical areas.

Furthermore, most papers deal with the problem of comparing the RR data resulting from satellite signal attenuation with those coming from conventional rain gauges. In general, these two rain measuring techniques are not exactly comparable, even when the rain gauge is placed in close proximity to the satellite receiver. This is true in view of at least two types of arguments: on the one hand, the variation of the received signal power with respect to the baseline is related to the instantaneous distribution of RR present on the wet segment of the radio path, while the rain gauge by its nature provides a measure of the cumulated (integrated) rainfall over a certain (albeit possibly short) period of time. It is noted that in order to render the two measurements homogeneous, many authors refer to cumulative pluviometric readings carried out at a fixed time spacing (e.g., one every 5′) and to compare them with the instantaneous RR measurements produced by the satellite receiver integrated for the same period of time.

On the other hand, the rain gauge produces point-scale measurements, i.e., it provides information about the rain actually fallen during the intended time interval precisely at the rain gauge site while the power reduction experienced by the GS is the overall effect of rain on the entire wet segment of the satellite–GS radio path, along which the RR is not necessarily constant; therefore, it results in a sort of “average” RR on the wet segment.

The different inherent operations of the above two sensors may sometimes result in conflicting indications about rainfall presence and intensity. For example, it may happen that the satellite receiver perceives a signal level reduction when it is not yet raining over the GS site, and vice versa the rain gauge may start detecting raindrops when the precipitation does not yet significantly impact the received power. In any case, experience shows that the above effects can be ignored to a first approximation when the horizontal projection of the radio path wet segment (for which its length is $L_{s}\cos\theta$ from Figure 1) is of the same order or shorter than the rain cell extension, which is after all a rather common situation. In any case, the impact of non-uniform rain distribution on the performance of the satellite system can be mitigated through application of adequate corrective coefficients when mapping the measured rain attenuation $L_{\mathrm{rain}}$ on an estimate of RR (Section 4.2.3).

Further improvements of the RR estimation accuracy in the vicinity of the receiver site can be achieved if it is possible to build a 2D geographic map of the rain field through joint processing of the data collected from multiple terminals scattered throughout the area. This type of approach permits, in fact, evaluating the RR gradient on the wet segment of the radio path.

#### 5.3.4. Latency Issues

Another aspect in which the proposed algorithms may significantly differ from one another is their capability to produce the RR data in (almost) real time. This issue is important in view of the possible use of these techniques in support of surveillance and early alert infrastructure deployed to counteract potentially dangerous weather events. Some authors demonstrate specific awareness and attention to this problem: for example, the approach discussed in [5,54] is based on a neural network purposely designed for the detection of rain with a negligible delay so as to meet the requirements of real-time operation. Rather curiously, in order to obtain the SNR baseline during a rain event, the algorithms use interpolation of the dry-state baseline measured at the start and stop of rain, a requirement that seems hardly compatible with real-time operation. A similar observation can be made about the algorithm in [5], regarding the possible incompatibility with real-time operation of the prior removal of the periodic component from the input data. In many other papers [1,5] more focused on demonstrating the feasibility of RR estimation through the exploitation of a satellite link, the issue of latency is not central to the paper scope and is, therefore, ignored.

### 5.4. Ancillary Information Required by the RR Estimation Algorithm

#### 5.4.1. Rain Height vs. 0 °C isotherm Height

All of the algorithms discussed so far require knowledge of the rain height in order to determine the length of the wet segment on the radio path (Section 4.2.1). With reference to a stratiform precipitation, this height is closely related to the altitude $h_{0}$ of the 0 °C isotherm [61,62,63,64,65,66,67], which in its turn can be identified by three methods.

The first, and the most accurate one, consists in relying on an external source (typically a weather service agency) providing actual measurements of $h_{0}$ generated by radar sensors or, accepting a slightly lower performance, on short-term forecasts of $h_{0}$ made by a numerical weather prediction (NWP) method, such as the Weather Research and Forecasting (WRF) model developed by LaMMA Consortium based in Florence, Italy (https://www.lamma.rete.toscana.it/en (accessed on 28 August 2021)).

The second method consists in setting $h_{0}$ as the daily average obtained from some public-domain database containing the recordings of the 0 °C isotherm height over a significantly large number of years for the site where the receiver is installed, such as NASA’s Modern-Era Retrospective analysis for Research and Applications (MERRA) database (https://gmao.gsfc.nasa.gov/reanalysis/MERRA (accessed on 28 August 2021)) [68]. This average/typical value of $h_{0}$ is then used to identify the rain height $h_{R}-h_{S}$ (see also discussion in Section 3.2), which finally through substitution in (11)–(12) permits calculating the specific attenuation. This method sacrifices some performance since it is clearly affected by errors resulting from the random fluctuations of the actual value of $h_{0}$ with respect to the statistical data reported in the database. However, it greatly simplifies the implementation as it requires only the availability of the site-specific, pre-calculated set of 365 daily averages, without any connection to external databases.

A third method, although a rather crude approximation, consists in assigning a constant value to $h_{0}$ given by the yearly average of the 0 °C isotherm height, provided from ITU-R’s recommendation P.839-4 (https://www.itu.int (accessed on 28 August 2021)). Clearly, this method further sacrifices performance, but it is also the easiest to implement as it requires only the knowledge of a constant, site-specific value.

Once the value of $h_{0}$ has been identified, it is possible to proceed to the determination of the rain height to be used in (11)–(12) for the calculation of the specific attenuation. Actually, the rain height rarely coincides with $h_{0}$ in view of the existence of the ML, where ice particles coexist with raindrops, which itself is responsible for part of the signal attenuation. As pointed out in Section 3.2, in order to obtain an accurate estimate of the specific attenuation to be ascribed only to the liquid phase layer, it would be necessary to know both the attenuation introduced globally by the ML (which attenuates in a different way compared to the LL) and its vertical thickness (usually in the order of hundreds of meters) to be subtracted from $h_{0}$, and this task appears quite challenging in terms of type, number and cost of weather sensors required.

A less accurate model but easier to implement is to consider the ML absent, adopting an equivalent liquid rainfall height (usually higher than the real one), to which the entire attenuation of the signal is ascribed, including the suppressed ML. This type of approach, preferred by most authors, is also suggested in [52], where it is recommended to assume an equivalent rain height equal to $h_{0}$ augmented by the fixed amount of 0.36 km. There are also some papers [69] in which the rain height is assumed to coincide with that of the lower edge of the clouds that can be measured with weather sensors.

#### 5.4.2. Impact of the Melting Layer

Some papers investigate in greater detail the impact of the ML (see Section 3.2.2 and Section 4.2.2) by proposing models to calculate its contribution to the attenuation, as distinct from that due to the entirely liquid layer. Papers [32,34], which are widely cited in the literature, proposed to treat the ML in the same manner as the LL by fictitiously characterizing it by a constant RR equal to that in the LL as the altitude varies, with specific attenuation still given by an expression of the type (5), albeit with coefficients *a* and *b* different from those used in the LL and to generate the actual attenuation of the ML. In [8], a variant of this method is proposed wherein the specific attenuation model (5) is applied to elementary horizontal slices of ML, characterized by a liquid fraction varying linearly with altitude as in one of the models assumed in [32], starting from 0 mm/h at $h_{0}$ and ending up to the full RR value of the LL at the lower edge of the ML. As for the ML vertical thickness, it is obtained either from measurements carried out by means of a weather sensor [8] or provided by some short-term weather forecasting agency or, again, drawn from a statistical database as an average or typical value. During an experimental campaign carried out in Rome (Italy) [70], the signature of the melting layer was obtained with a dual-polarization radar. Polarimetric measurements at vertical incidence showed that the vertical extent of the ML ranges between 150 and 600 m. Accordingly, in the practical implementation of the Nefocast algorithm for RR estimation, the thickness of the ML was assumed as 500 m. To be finally noted, in [8] it is shown that this RR estimation technique is weakly affected by (even large) errors in the knowledge of the ML thickness.

### 5.5. Sudden Variations of Signal Level Due to Causes Different from Rain

#### 5.5.1. Sun Blinding

The sun blinding effect consists of an abrupt growth of the noise level due to the passage of the sun near the pointing direction of the GS antenna and is mentioned in Section 3.1.3 as one of the possible impairments of the downlink, occasionally being so severe as to cause link outage. However, due to its marginal and sporadic impact on the signal quality, it is substantially disregarded in the references of interest here. As already noted, the solar outage events are deterministic (although their intensity and duration depend on the gain of the ground antenna, i.e., on the angular width of the main lobe in its radiation pattern), and signal segments affected by this phenomenon can be predicted very accurately, allowing momentarily suspending the RR detection and estimation algorithms in these intervals. The gaps that these events leave in the sequence of measurements have a short duration (a few minutes); thus, it seems reasonable in the meantime to keep the dry baseline fixed at the last value assumed prior the sun transit for the whole duration of the fade event, as suggested in [8]. It is also observed that, during a sun transit, both the received power and the SNR vary in a smooth and almost deterministic manner (see Figure 3); thus, it can be argued that automatic rain detection algorithms based on the analysis of the measurement statistics, such as for example those who use neural networks, would be able to correctly classify the event as non-rainy.

#### 5.5.2. Operator Corrections

This impairment factor is mentioned in Section 3.1.4. Similarly to sun blinding events, the operator’s corrections of satellite transmission and satellite kinematic parameters may entail large and sudden variations of the received power, but unlike sun passages the latter kind of events are not predictable in terms of time of occurrence nor in their intensity and (increase/decrease) sign. However, in order to recognize this type of events, it seems possible to resort to techniques already mentioned in the previous section, based on the use of a neural network for the classification of signal statistics. These types of techniques, as already observed, have been proposed to recognize the rain/no rain state by analyzing the behavior of the received signal [5]: If the neural network, following a sudden change in the signal level, does not recognize the statistical behavior typically induced by rain, after a relatively short time it classifies the event as a false alarm and returns the RR estimation algorithm back to dry state operation. A similar approach, whereby a decision about the presence or absence of rain is taken following an evaluation of the statistical behavior of the signal, is also pursued in [8]. Furthermore, following [8] it is worth remarking that signal variations induced by the satellite operator maneuvers can be easily recognized if there are several GSs belonging to a regional network, all equipped with return links towards a single data processing center. In such a case, in fact, signal variations occur simultaneously for all the terminals; therefore, they can be identified and processed globally at a central level.

## 6. Conclusions

In this survey paper, we have shown that in recent years—roughly in the last two decades—considerable interest has been devoted to novel low-cost techniques for the early detection and evaluation of precipitations, relying on the opportunistic use of existing satellite networks, notably those based on geostationary satellites intended for domestic broadcast and internet access services. The underlying principle is conceptually simple although not necessarily easy to implement, and it is based on the measurement of the attenuation introduced by the rain on the downlink signal and on the application of a proper inversion algorithm, appropriately geared to map this attenuation onto a rainfall rate estimate. The above techniques have been studied with the aim to integrate or even replace the pre-existing infrastructures based on rain gauges, radar sensors and meteorological satellites, with respect to which this new approach prospectively offers greater pervasiveness and capillarity over the territory thanks to the potentially huge number of terrestrial terminals deployable in households, each amenable to act as a rain sensor. Another peculiar feature of these systems is that of presenting an estimation accuracy independent of the site at which the terrestrial terminal is positioned, unlike weather radars for which its accuracy in providing estimates of instantaneous point-scale rain rate becomes poorer and poorer as the distance from the radar increases.

The material we have presented comes from the elaboration of a significant body of literature on the above topics. Since the cited opportunistic approach has been proposed in support or as an alternative to traditional rain sensing techniques, most papers delve into the relative performance of specific implementations of the new method with respect to rain gauges and weather radars, notably with regard to their ability to measure the instantaneous rainfall rate and the cumulated rainfall at a given geographical site. In this respect, one point of strength of the systems based on downlink attenuation measurements is their sensitivity to the instantaneous rainfall rate, and this provides them with an intrinsic aptitude to operate in real-time alert networks, while rain gauges can only detect the cumulated rainfall in a certain time interval. On the other hand, rain gauges yield measurements of rain that has fallen at the exact geographical site where they are deployed, while systems measuring the downlink signal attenuation are sensitive to the effect of the rain over the entire “wet” section of the radio path between the satellite and the terrestrial receiver, and therefore are not strictly suited for providing point-scale measurements. However, this apparently poor compatibility of the new opportunistic with respect to traditional sensors has been resolved by the various authors by resorting to a variety of ad hoc techniques discussed in the paper.

The advantageous use of the opportunistic sensors requires several collateral problems be addressed and solved. In this paper we have identified, enumerated and discussed an exhaustive selection of these issues, and we have illustrated a number of solutions proposed in the literature while also highlighting their relative points of strength and weakness. In summary, the issues to deal with for the implementation of a rain sensor based on measurements of satellite downlink attenuation are substantially related to the following: (i) identification of type of satellites and bands to be used; (ii) identification of the type of downlink signal, whether payload or beacon, on which to measure the rain-induced attenuation while also taking into account the impact of this choice on the complexity and cost of the terrestrial receiver; (iii) choice to be made between the point-scale approach in which each measurement of attenuation produced by a single terrestrial receiver is mapped directly onto an estimate of the local rainfall rate and the tomographic approach in which the rainfall rate at each point of the territory is estimated by processing several attenuation measurements collected from multiple radio links intersecting the rain field along different directions; (iv) identification of an efficient algorithm for detecting the instants of rain start and rain stop; (v) identification of methods for estimating or monitoring the height of the rain volume and the thickness of the melting layer, along with their respective specific attenuation coefficients; (vi) identification of the time-varying baseline for evaluating the rain-induced reduction in the relevant signal metric, either the SNR or the ESNDR; and (vii) the evaluation of the algorithm latency and its compatibility with real-time operation.

With regard to the state of the art and future perspectives of the techniques covered in this paper, it is noted that most of the algorithms proposed are still in the experimental phase, while only a few, such as those in [3,8] and [6,48], have been brought to a more advanced level of validation and pre-engineering development.

In this respect, it cannot be ignored that a decisive factor for a widespread adoption of these techniques is the existence on the market of commercial devices suited to facilitate their opportunistic usage. One instance is the SmartLNB, a bidirectional device designed for domestic satellite TV which, as illustrated in Section 5.2.2, not only easily lends itself to measuring the ESNDR on the downlink signal but also allows measurement results and other processed data to be transmitted back to a control station via a satellite return channel with no need for any additional terrestrial infrastructure. We observe that such a particular use of these devices could be currently considered at risk of decline due to the rapid spread of internet TV platforms. On the other hand, the impetuous growth expected in the near future for satellite IoT prospectively guarantees many further years of bright future for interactive domestic satellite terminals.

Finally, we note that satellites could represent an excellent complementary technology to fill the coverage gaps of rain monitoring networks based on the (far more mature) opportunistic use of terrestrial microwave links [11], as testified by some very promising preliminary results presented in [71].

## Figures and Tables

**Table 1:** Frequency bands for the downlink of communication satellites above 10 GHz (ITU Region 1).

Band	Frequency (GHz)	Service	Direction	Ref.
Ku	10.70–12.75	Space-to-Earth, Fixed-Satellite: Broadcasting-satellite service	downlink	[19]
Ka	17.30–20.20	Space-to-Earth, Fixed-Satellite: Broadband satellite service	downlink	[20]
Ka	21.40–22.00	Space-to-Earth, Fixed-Satellite: Broadcasting-satellite service	downlink	[21]
Q/V	37.50–43.50	Space-to-Earth, Fixed-Satellite: Broadband satellite service	downlink	[22]

**Table 2:** List of geostationary satellites for DTH TV broadcasting in the Ku band Table 10 (4147° E, 43.7117° N).

Geostationary Satellite	Long (°)	Azimuth w.r.t. Local South (°)	Elevation above Horizon (°)	Beam	EIRP (dBW)	Reference
Eutelsat 70B	70.5	−60.0853	12.6724	Wide	44	LyngSat. Available online: https://www.lyngsat.com (accessed on 28 August 2021).
Intelsat 20	68.5	−58.0853	14.0569	Europe and Africa	44.8	LyngSat. Available online: https://www.lyngsat.com (accessed on 28 August 2021).
Intelsat 17	66.0	−55.5853	15.7709	Europe and Mid-East	47.6	Digisat. Available online: https://www.digisat.org (accessed on 28 August 2021).
NSS 12	57.0	−46.5853	21.7379	Middle East	46	LyngSat. Available online: https://www.lyngsat.com (accessed on 28 August 2021).
Express AM6	53.0	−42.5853	24.2543	Fixed 2	47	LyngSat. Available online: https://www.lyngsat.com (accessed on 28 August 2021).
Belintersat 1	51.5	−41.0853	25.1708	Europe	51	Belintersat. Available online: https://belintersat.com (accessed on 28 August 2021).
Türksat 4B	50.0	−39.5853	26.0710	West	54	SatStar. Available online: http://www.satstar.net (accessed on 28 August 2021).
AzerSpace 1/Africasat 1a	46.0	−35.5853	28.3825	Europe	46	SatStar. Available online: http://www.satstar.net (accessed on 28 August 2021).
AzerSpace 2/Intelsat 38	45.1	−34.6853	28.8831	Europe	51.6	LyngSat. Available online: https://www.lyngsat.com (accessed on 28 August 2021).
Türksat 3A/Türksat 4A	42.0	−31.5853	30.5469	West	52	LyngSat. Available online: https://www.lyngsat.com (accessed on 28 August 2021).
Hellas Sat 3/Hellas Sat 4	39.0	−28.5853	32.0591	Europe FSS	52	SatStar. Available online: http://www.satstar.net (accessed on 28 August 2021).
Eutelsat 36B	36.0	−25.5853	33.4642	Eurasia	48	LyngSat. Available online: https://www.lyngsat.com (accessed on 28 August 2021).
Eutelsat 33E	33.0	−22.5853	34.7513	Wide	50	SatStar. Available online: http://www.satstar.net (accessed on 28 August 2021).
Astra 5B	31.5	−21.0853	35.3471	High power	52	LyngSat. Available online: https://www.lyngsat.com (accessed on 28 August 2021).
Astra 2E/Astra 2F/Astra 2G	28.2	−17.7853	36.5375	Europe	50	LyngSat. Available online: https://www.lyngsat.com (accessed on 28 August 2021).
Badr 4	26.0	−15.5853	37.2336	BSS	47	Arabsat. Available online: https://www.arabsat.com (accessed on 28 August 2021).
Astra 3B	23.5	−13.0853	37.9242	Europe	51	LyngSat. Available online: https://www.lyngsat.com (accessed on 28 August 2021).
Eutelsat 21B	21.5	−11.0853	38.3962	Western	46	LyngSat. Available online: https://www.lyngsat.com (accessed on 28 August 2021).
Astra 1KR/Astra 1L/Astra 1M/Astra 1N	19.2	−8.7853	38.8473	Europe	51	LyngSat. Available online: https://www.lyngsat.com (accessed on 28 August 2021).
Eutelsat 16A	16.0	−5.5853	39.3062	Europe B	50	LyngSat. Available online: https://www.lyngsat.com (accessed on 28 August 2021).
Hotbird 13B/Hotbird 13C/Hotbird 13E	13.0	−2.5853	39.5528	Wide	53	LyngSat. Available online: https://www.lyngsat.com (accessed on 28 August 2021).
Eutelsat 10A	10.0	0.4147	39.6185	Wide	48	LyngSat. Available online: https://www.lyngsat.com (accessed on 28 August 2021).
Eutelsat 9B	9.0	1.4147	39.6000	Wide	51	LyngSat. Available online: https://www.lyngsat.com (accessed on 28 August 2021).
Eutelsat 7B/Eutelsat 7C	7.0	3.4147	39.5026	Europe A	49	Eutelsat. Available online: https://www.eutelsat.com (accessed on 28 August 2021).
Astra 4A	4.9	5.5147	39.3140	Europe BSS	50	LyngSat. Available online: https://www.lyngsat.com (accessed on 28 August 2021).
Eutelsat 3B	3.0	7.4147	39.0683	Europe	49	LyngSat. Available online: https://www.lyngsat.com (accessed on 28 August 2021).
BulgariaSat 1	1.9	8.5147	38.8938	Europe	50	LyngSat. Available online: https://www.lyngsat.com (accessed on 28 August 2021).
Thor 7/Thor 5/Thor 6/Intelsat 10-02	−0.8	11.2147	38.3679	Spot 1	53.2	LyngSat. Available online: https://www.lyngsat.com (accessed on 28 August 2021).
ABS 3A	−3.0	13.4147	37.8395	Europe	50	ABS. Available online: https://absatellite.com (accessed on 28 August 2021).
Amos 7/Amos 3	−4.0	14.4147	37.5706	Pan European	53	Spacecom. Available online: https://www.amos-spacecom.com (accessed on 28 August 2021).
Eutelsat 5 West A (incl. 1.3°)/Eutelsat 5 West B	−5.0	15.4147	37.2842	Super	53	LyngSat. Available online: https://www.lyngsat.com (accessed on 28 August 2021).
Eutelsat 8 West B	−8.0	18.4147	36.3237	Europe	54	SatStar. Available online: http://www.satstar.net (accessed on 28 August 2021).
Express AM44	−11.0	21.4147	35.2191	S1	51	LyngSat. Available online: https://www.lyngsat.com (accessed on 28 August 2021).
Express AM8	−14.0	24.4147	33.9811	Fixed 1	51	LyngSat. Available online: https://www.lyngsat.com (accessed on 28 August 2021).
Telstar 12 Vantage	−15.0	25.4147	33.5407	Europe and Middle East	51	SatStar. Available online: http://www.satstar.net (accessed on 28 August 2021).
NSS 7 (incl. 4.9°)	−20.0	30.4147	31.1490	Europe and Middle East	49	SatStar. Available online: http://www.satstar.net (accessed on 28 August 2021).
SES 4	−22.0	32.4147	30.1113	Europe and Middle East	49	LyngSat. Available online: https://www.lyngsat.com (accessed on 28 August 2021).
Intelsat 905 (incl. 2.0°)	−24.5	34.9147	28.7563	Spot 1	52.6	LyngSat. Available online: https://www.lyngsat.com (accessed on 28 August 2021).
Intelsat 901	−27.5	37.9147	27.0528	Spot 2	45	SatStar. Available online: http://www.satstar.net (accessed on 28 August 2021).
Hispasat 30W-5/Hispasat 30W-6	−30.0	40.4147	25.5753	Europe	54	LyngSat. Available online: https://www.lyngsat.com (accessed on 28 August 2021).
Telstar 11N	−37.5	47.9147	20.8807	Europe	52	SatStar. Available online: http://www.satstar.net (accessed on 28 August 2021).

**Table 3:** Parameters of a DTH TV broadcast satellite downlink [8]. LVP: linear vertical polarization. QPSK: quadrature phase shift keying. DVB-S2: digital video broadcasting via satellite, 2nd generation. FEC: forward error correction. QEF: quasi error free, refers to PER =10^−7^ (see [20], Table 13). PER: packet error rate.

Receiving location	Pisa, Italy (10.4147° E, 43.7117° N)
Satellite name; orbital slot; inclination	Eutelsat 10A; 10° East; 0.072°
Satellite EIRP	48 dBW
Frequency; polarization	11.345 GHz; LVP
Protocol; modulation; FEC rate	DVB-S2; QPSK; 4/5
Required ESNDR for QEF performance	4.68 dB
Figure of noise of the receiver	0.2 dB @ 290 K
Noise temperature of the receiver, $T_{\mathrm{RX}}$	13.7 K
Atmospheric attenuation, $L_{\mathrm{atm}}$	0.09 dB
Mean thermodynamic temperature of troposphere, $T_{\mathrm{atm}}$	275 K
Noise temperature of the CBMR, $T_{c}$	2.78 K
Noise spillover temperature, $T_{g}$	10 K
Receiving antenna diameter; gain (60% efficiency)	80 cm; 37.34 dBi
Typical received ESNDR in clear-sky conditions $E_{s}/N_{0}$	10.5 dB
Link margin	5.82 dB
θ	40°

**Table 4:** Parameters of a beacon-receiving system using a Ka-band broadband satellite: (**a**) system specifications (based on the numerical values used in [47]); (**b**) performance.

(**a**)			
a.1	Beacon frequency		20 GHz (Ka Band)
a.2	Beacon EIRP		30 dBW
a.3	B		17 Hz
a.4	$L_{\mathrm{FS}}$		210 dB
a.5	$L_{\mathrm{atm}}$		1.0 dB
a.6	$T_{\mathrm{atm}}$		275 K
a.7	$T_{c}$		2.78 K
a.8	$T_{g}$		10 K
a.9	$T_{\mathrm{RX}}$		100 K
a.10	$G_{R}$		40 dBi
(**b**)			
Clear-sky, $L_{\mathrm{rain}}$ = 0 dB			
b.1	C	−111.00 dBm	Equation (1)
b.2	N	−164.35 dBm	Equations (2)–(4)
b.3	C/N	53.35 dB	(b.1), (b.2)
b.4	C+N	−111.00 dBm	(b.1), (b.2)
b.5	(C+N)/N	53.35 dB	(b.4), (b.2)
Rain, $L_{\mathrm{rain}}$ = 44 dB			
b.6	C	−155.00 dBm	Equation (1)
b.7	N	−160.44 dBm	Equations (2)–(4)
b.8	C/N	5.44 dB	(b.6), (b.7)
b.9	C+N	−153.91 dBm	(b.6), (b.7)
b.10	(C+N)/N	6.53 dB	(b.9), (b.7)
